# Acute Effects of High-Definition Transcranial Direct Current Stimulation on Foot Muscle Strength, Passive Ankle Kinesthesia, and Static Balance: A Pilot Study

**DOI:** 10.3390/brainsci10040246

**Published:** 2020-04-21

**Authors:** Songlin Xiao, Baofeng Wang, Xini Zhang, Junhong Zhou, Weijie Fu

**Affiliations:** 1 School of Kinesiology, Shanghai University of Sport, Shanghai 200438, China; xiao_songlin@126.com (S.X.); wangbaofeng1911@163.com (B.W.); zhangxini1129@163.com (X.Z.); 2 The Hinda and Arthur Marcus Institute for Aging Research, Hebrew SeniorLife, Boston, MA 02131, USA; 3 Harvard Medical School, Boston, MA 02131, USA; 4 Key Laboratory of Exercise and Health Sciences of Ministry of Education, Shanghai University of Sport, Shanghai 200438, China

**Keywords:** high-definition transcranial direct current stimulation (HD-tDCS), foot muscle strength, passive ankle kinesthesia, static balance

## Abstract

This study aimed to examine the effects of single-session anodal high-definition transcranial direct current stimulation (HD-tDCS) on the strength of intrinsic foot muscles, passive ankle kinesthesia, and static balance. Methods: In this double-blinded self-controlled study, 14 healthy younger adults were asked to complete assessments of foot muscle strength, passive ankle kinesthesia, and static balance before and after a 20-minute session of either HD-tDCS or sham stimulation (i.e., control) at two visits separated by one week. Two-way repeated-measures analysis of variance was used to examine the effects of HD-tDCS on metatarsophalangeal joint flexor strength, toe flexor strength, the passive kinesthesia threshold of ankle joint, and the average sway velocity of the center of gravity. Results: All participants completed all study procedures and no side effects nor risk events were reported. Blinding was shown to be successful, with an overall accuracy of 35.7% in the guess of stimulation type (*p* = 0.347). No main effects of intervention, time, or their interaction were observed for foot muscle strength (*p* > 0.05). The average percent change in first-toe flexor strength following anodal HD-tDCS was 12.8 ± 24.2%, with 11 out of 14 participants showing an increase in strength, while the change following sham stimulation was 0.7 ± 17.3%, with 8 out of 14 participants showing an increase in strength. A main effect of time on the passive kinesthesia threshold of ankle inversion, dorsiflexion, and anteroposterior and medial–lateral average sway velocity of the center of gravity in one-leg standing with eyes closed was observed; these outcomes were reduced from pre to post stimulation (*p* < 0.05). No significant differences were observed for other variables between the two stimulation types. Conclusion: The results of this pilot study suggested that single-session HD-tDCS may improve the flexor strength of the first toe, although no statistically significant differences were observed between the anodal HD-tDCS and sham procedure groups. Additionally, passive ankle kinesthesia and static standing balance performance were improved from pre to post stimulation, but no significant differences were observed between the HD-tDCS and sham procedure groups. This may be potentially due to ceiling effects in this healthy cohort of a small sample size. Nevertheless, these preliminary findings may provide critical knowledge of optimal stimulation parameters, effect size, and power estimation of HD-tDCS for future trials aiming to confirm and expand the findings of this pilot study.

## 1. Introduction

A new paradigm has redefined the complex human foot structure as the foot core system, which includes the active, passive, and neural subsystems [1]. The active subsystem is composed of intrinsic and extrinsic foot muscles that can control foot movement and provide propulsive power, while the neural subsystem comprises sensory receptors that provide accurate motion sensory messages regarding ankle posture [2]. The active and neural subsystems are important in maintaining standing balance and controlling body posture [3]. Impaired movement sense and reduced foot muscle strength increase walking variability, fall risk [4,5], and even sports-related injuries such as plantar fasciitis and chronic ankle instability (CAI) [6,7]. Therefore, many studies have focused on strengthening the foot core system to prevent foot injuries. To our knowledge, previous studies have mainly focused on enhancing foot function and preventing foot injuries by strengthening intrinsic foot muscles and peripheral nervous systems [1,2]. However, the central nervous system plays a critical role in altering motor planning and generating movement patterns, and changes within the central nervous system predispose individuals to re-injury [8]. Decreased excitability of the primary motor cortex (M1) and reduced activation of the somatosensory cortex (S1) have been reported in individuals with foot injuries, e.g., CAI [8]. Therefore, strategies designed to target the cortical sensorimotor regions of the brain hold great promise for improving functional performance pertaining to the foot, and may thus help prevent foot-related injuries in sports.

Transcranial direct current stimulation (tDCS) is a safe method for modulating the excitability of brain regions noninvasively by inducing a low-amplitude current flow between two or more electrodes placed on the scalp [9]. Several systematic reviews and meta-analyses have demonstrated that anodal tDCS applied over M1 can improve balance control, promote muscle strength and muscular endurance, and enhance exercise performance in cycling [10,11,12]. Studies have also shown that anodal tDCS designed to target the sensorimotor regions of the brain improves physical performance, including muscle strength and sensory function [13]. Specifically, researchers observed that one session of tDCS targeting M1 enhanced the isometric strength of quadricep femoris [14] and the toe pinch force [15]. Zhou et al. [16] recently observed that single-session tDCS over S1 induced the improvement of vibrotactile sensation of the foot sole of older adults under weight-bearing conditions. These studies suggested that anodal tDCS can improve muscle strength and foot sole somatosensation by increasing the cortical excitability of the sensorimotor regions of the brain.

However, these studies used conventional tDCS with large sponge electrodes. This may cause a tingling sensation over the scalp. Moreover, the results indicated large interpersonal variance, which may be due to the current delivered by conventional tDCS diffusing in the cortical regions [17]. Fortunately, novel high-definition tDCS (HD-tDCS) has been developed by employing advanced neuro-modeling techniques; small electrodes enable the navigation of current flow in cortical regions and thus induce a “focal” electric field on targets [18]. The effects of this HD-tDCS technique on human motion function and performance, however, have not been explored. We here anticipate that anodal HD-tDCS can be used as an effective approach to improve foot muscle strength, ankle kinesthesia, and balance performance pertaining to these functions.

This study aimed to examine the effects of single-session anodal HD-tDCS on the strength of foot plantar muscles, passive ankle kinesthesia, and static balance ability. We hypothesized that compared to sham stimulation (i.e., control), single-session anodal HD-tDCS could enhance metatarsophalangeal joint (MPJ) flexor and toe flexor strength, decrease the passive kinesthesia threshold of the ankle joint, and improve static balance ability in healthy younger adults.

## 2. Methods

### 2.1. Participants

Fourteen healthy young male adults (age: 22.8 ± 1.2 years; height: 174.6 ± 6.6 cm; body mass: 72.2 ± 8.8 kg; dominant leg: right, as defined by the preferred kicking leg [19]) were recruited. The sample size was calculated using a power analysis with a statistical power of 0.80, a probability level of 0.05, and an effect size f of 0.38 [20] via G*Power 3.1.9.2 software [21,22]. The analysis gave a sample of 11 participants. Considering a 20% drop-out rate, 14 participants were recruited in this study. Participants were recruited from a university community through the distribution of flyers and email announcements of the study. The inclusion criteria were as follows: (1) good health of participants in terms of normal muscle strength and sensory function and (2) no history of lower extremity injuries in the past 6 months. Those who had skin allergies, were using neuropsychiatric medication, had a major neurological disease, or had any contraindications with respect to the use of tDCS (e.g., metal-implanted devices in the brain) were excluded. The participants were asked not to engage in strenuous exercises within 24 h prior to testing and not to drink any beverages containing stimulants such as caffeine within 4 h prior to testing to limit the potential influence of heavy-load physical activity or caffeine on their performance. All participants provided a written informed consent as approved by the Institutional Review Board of the Shanghai University of Sport (2019RT020).

### 2.2. Experimental Protocol

In this randomized double-blinded, self-controlled study, each participant completed two visits consisting of functional tests (i.e., passive ankle kinesthesia, foot muscle strength, and static balance) immediately before and after a 20-minute session of either HD-tDCS or sham stimulation in a randomized order. The tests started at the same time of a day on each visit, and the two visits were separated by one week to largely eliminate the after-effects of stimulation and to diminish repetition effects. All participants completed the tests in the same order: passive ankle kinesthesia first, then foot muscle strength, and finally static balance. Between different types of tests, a 5-min break was provided to eliminate the effects of fatigue on task performance.

### 2.3. High-Definition Transcranial Direct Current Stimulation Intervention

**Figure 1:**
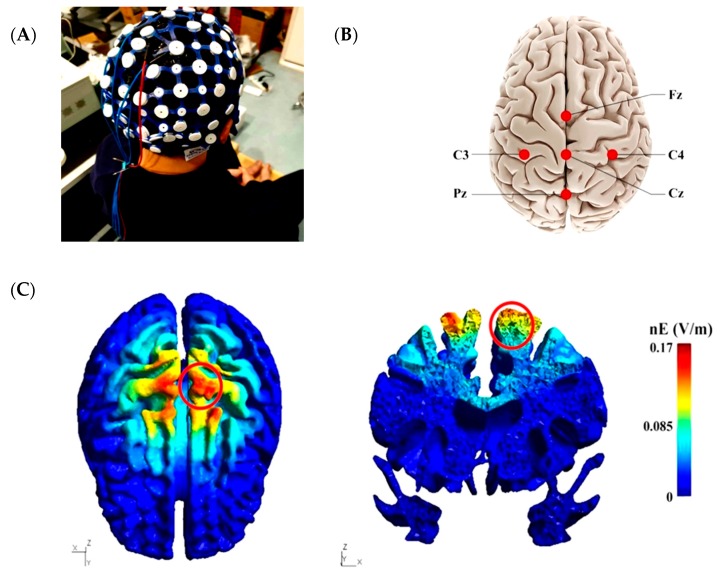
High-definition transcranial direct current stimulation (HD-tDCS) electrode placement and electrical current flow model. (**A**) The experimental setup for HD-tDCS. (**B**) Placement of 4 × 1 HD-tDCS electrodes. The anodal electrode was placed over the Cz electrode of a 10/20 electroencephalogram (EEG) system and surrounded by four cathodal electrodes i.e., C3, C4, Fz, and Pz. (**C**) Electrical current flow model of the cortical surface (left), and the cortical cross-section (right). The electrical field influenced the lower limb area of the sensorimotor regions (red circle).

The DC-STIMULATOR PLUS (neuroConn, Ilmenau, Germany) device was used to connect to a 4 × 1 multichannel stimulation adapter. The tDCS montage was designed to increase the lower limb area of the sensorimotor regions, i.e., M1 and S1. Five silver chloride-sintered circular electrodes with size of 1 cm^2^ were used. The anodal electrode was placed over the Cz electrode of a 10/20 electroencephalogram (EEG) system and was surrounded by four cathodal electrodes (each at a ring center-to-ring center distance of 3.5 cm from the anodal electrode, i.e., C3, C4, Fz, and Pz) (Figure 1A–C) [23]. HD-tDCS was administered for 20 min continuously at a target current intensity of 2.0 mA. This dose of HD-tDCS could exert prominent, long-lasting excitatory following stimulation. Moreover, this intensity has been proven to be safe and well-tolerated by participants [24,25]. Anodal HD-tDCS was applied with an electric current intensity of 2 mA for 20 min. In the anodal HD-tDCS, the current was ramped up to 2 mA over 30 s at 0.1-mA intervals. After 20 min of stimulation, the current was then ramped down to 0 mA over 30 s. In the sham stimulation, the parameters were the same as those in HD-tDCS, but the current was ramped up to 2 mA over 30 s and then immediately ramped down to 0 mA. According to previous studies, this provided enough time to identify the presence of the current with no effective brain stimulation [26,27]. The type of stimulation (i.e., HD-tDCS or sham) was programmed using a code only known by personnel uninvolved in any study procedure before the stimulation. Thus, neither the participants nor the study personnel knew the stimulation type (double-blinded method). The participants were asked to complete a questionnaire at the end of each stimulation to evaluate the potential side effects. They were also asked to “guess” whether they had received HD-tDCS or sham stimulation to assess the blinding efficacy.

### 2.4. Data Collection

#### 2.4.1. Passive Ankle Kinesthesia

The passive kinesthesia threshold of the ankle joint was assessed by using an ankle proprioception tester (KP-11, Toshimi, Shandong, China). The test–retest reliability of this instrument was verified with an intraclass correlation coefficient in range of 0.737–0.935 [28]. Each participant sat on an adjustable seat, and their hip, knee, and ankle joints were fixed at 90°. They each wore an eye mask and noise reduction earphones during the test. The dominant foot was bare, and the sole was wrapped with an air cushion to remove any tactile sense. The dominant foot was then relaxed and placed on the bottom of the foot pedal. Only half the weight of the lower extremity was loaded onto the platform. The platform was randomly activated to drive the participant’s ankle in plantarflexion (PF), dorsiflexion (DF), inversion (INV), and eversion (EV). Each participant was then instructed to complete at least three familiarity tests in each direction of ankle motion (i.e., PF, DF, INV, and EV). After confirming the trigger and the direction of foot movement, the participant was asked to press the stop button. The experimenter then recorded the angular displacement and movement direction. The participant lifted his foot from the platform, and the experimenter reset the instrument. After the familiarization test, the participant completed three trials of the test in each movement direction (i.e., PF, DF, INV, and EV) in a randomized order. A rest period of 1 min was given between trials.

#### 2.4.2. Metatarsophalangeal Joint Flexor Strength

**Figure 2:**
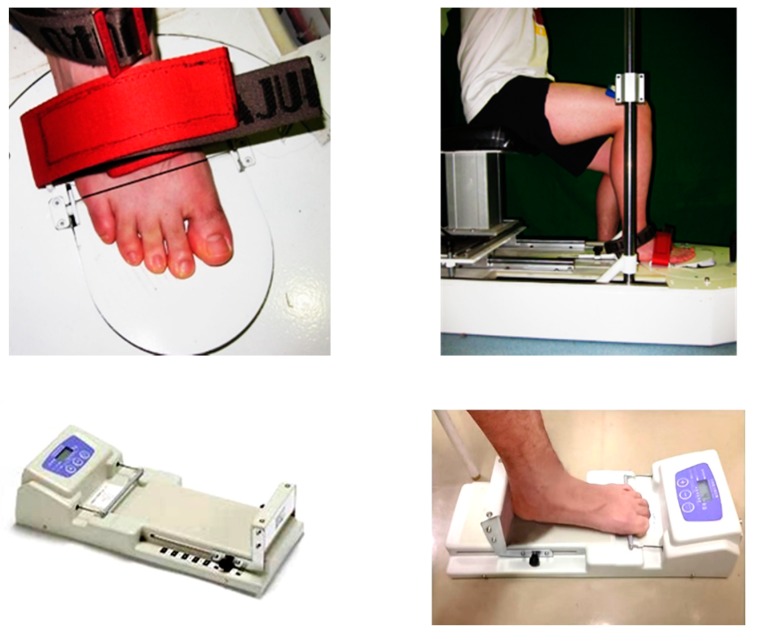
Metatarsophalangeal joint flexor strength tester (upper panels) and toe grip dynamometer and toe flexor strength measurement (lower panels).

MPJ flexor strength was measured using an MPJ flexor strength testing system customized by our team. The validity and reliability were reported previously [29,30]. Each participant was seated in the system with bare feet and legs. The position and height of the seat were adjusted to make the thighs parallel to the ground and the knee joint was fixed at 90°. The heels, ankles, and knees were fixed (Figure 2). When the test started, the participant was asked to flex the MPJ and press the pedal for 10 s with maximum force. The measurement was repeated thrice with a rest period of 1 min. The peak MPJ flexor strength was then obtained and normalized according to the body weight of each participant.

#### 2.4.3. Toe Flexor Strength

Toe flexor strength was measured in the sitting position using a toe grip dynamometer (T.K.K.3361, Takei Scientific Instruments Co., Niigata, Japan). Details of the tester, testing process, and its reliability are available in the literature [31,32]. Each participant was asked to sit on an adjustable seat, with the hip, knee, and ankle joints fixed at 90°. The dominant foot was placed on the dynamometer and fixed with the heel stopper, and the other foot was positioned next to the testing instrument. During the measurements, the toes were flexed vigorously for at least 3 s, and the trunk was kept upright while keeping the hands on the chest (Figure 2). The peak flexor strengths of the first toe, the other four toes, and all toes were recorded and normalized by body weight of each participant. The measurement was repeated thrice with an interval of 1 min.

#### 2.4.4. Static Balance Ability

In the standing balance test, each participant stood on the balance testing system (Super Balance, Acmeway, Beijing, China) while wearing a sports uniform (i.e., vest, shorts, and socks). While looking straight ahead, the participants stood in a position in which the width of their bare feet was the same as that of their shoulders. Each participant completed three trials in each of the following conditions: two-leg standing with eyes open (TL_EO) and eyes closed (TL_EC) and one-leg standing with eyes open (OL_EO) and eyes closed (OL_EC). Two-leg trials lasted 30 s, and one-leg trials lasted 10 s. A break of 30 s was provided between trials. The system recorded the sway velocity of the center of gravity (CoG) in the medial–lateral (ML) and anteroposterior (AP) directions.

### 2.5. Statistics

SPSS 22.0 (SPSS Inc., Chicago, IL, USA.) was used to complete the statistical analysis, and all data were expressed by mean ± standard deviation. The Shapiro–Wilk test was used to examine the normal distribution of the outcomes. Fisher’s exact test was used to test the blinding efficacy of HD-tDCS. Two-way repeated measures analysis of variance (ANOVA) was used to examine the main effects (intervention and time) and their interaction on functional performance. Post-hoc analysis was used if a significance in the interaction was observed. The significance level was set as *p* < 0.05. Effect size values ($\eta_{p}^{2}$) were reported for ANOVA.

## 3. Results

Fourteen participants received 2 mA of stimulation and completed all study procedures. No side effects or risk events were reported. For blinding efficacy, Fisher’s exact test showed a successful blinding procedure with an overall accuracy of 35.7% (*p* = 0.347).

The two-way repeated measures ANOVA revealed no significant intervention by time interaction effects for flexor strengths of the MPJ (*F*_(1, 26)_ = 0.472, *p* = 0.50, $\eta_{p}^{2}$ = 0.018), the first toe (*F*_(1, 26)_ = 3.124, *p* = 0.09, $\eta_{p}^{2}$ = 0.107), the other four toes (*F*_(1, 26)_ = 0.001, *p* = 0.97, $\eta_{p}^{2}$ < 0.001), and all five toes (*F*_(1, 26)_ = 0.547, *p* = 0.47, $\eta_{p}^{2}$ = 0.021). Further, no significant main effects of time and intervention were observed for any of these variables (*p* > 0.05). Specifically, the average percent change of the first-toe flexor strength following anodal HD-tDCS was 12.8 ± 24.2%, with 11 out of 14 participants showing an increase in strength, while the change following sham stimulation was 0.7 ± 17.3%, with 8 out of 14 participants showing an increase in strength.

No significant intervention by time interaction effects were observed for the passive kinesthesia thresholds of PF (*F*_(1, 26)_ = 0.329, *p* = 0.57, $\eta_{p}^{2}$ = 0.012), DF (*F*_(1, 26)_ = 0.158, *p* = 0.69, $\eta_{p}^{2}$ = 0.006), INV (*F*_(1, 26)_ = 0.072, *p* = 0.79, $\eta_{p}^{2}$ = 0.003), and EV (*F*_(1, 26)_ = 0.237, *p* = 0.63,$\;\eta_{p}^{2}$ = 0.009). A significant main effect of time was observed for the INV kinesthesia threshold (*F*_(1, 26)_ = 9.606, *p* = 0.005, $\eta_{p}^{2}$ = 0.270) and the DF kinesthesia threshold (*F*_(1, 26)_ = 5.409, *p* = 0.03, $\eta_{p}^{2}$ = 0.172), whereas no significance was observed in the main effects of the intervention. The INV and DF kinesthesia thresholds were significantly decreased after the stimulation as compared to pre-stimulation regardless of the two stimulation types (*p* < 0.05). Moreover, the INV kinesthesia threshold in 13 out of the 14 participants specifically decreased after anodal HD-tDCS, while this occurred in 8 out of the 14 participants after sham stimulation. The average percent decrease in the INV and DF kinesthesia thresholds following anodal HD-tDCS was 13.1 ± 17.6% (0.4 ± 0.4°) and 3.3 ± 17.1% (0.1 ± 0.3°), respectively, while the average percent change following sham stimulation was 9.4 ± 22.1% (0.3 ± 0.8°) and 7.4 ± 18.0% (0.2 ± 0.3°), respectively (Table 1).

The two-way repeated measures ANOVA revealed no significant intervention by time interaction effects for the ML average CoG sway velocity in TL_EO (*F*_(1, 26)_ = 0.250, *p* = 0.62, $\eta_{p}^{2}$ = 0.010), AP average CoG sway velocity in TL_EO (*F*_(1, 26)_ = 1.063, *p* = 0.312, $\eta_{p}^{2}$ = 0.039), ML average CoG sway velocity in TL_EC (*F*_(1, 26)_ = 1.056, *p* = 0.314, $\eta_{p}^{2}$ = 0.039), AP average CoG sway velocity in TL_EC (*F*_(1, 26)_ = 0.020, *p* = 0.89, $\eta_{p}^{2}$ = 0.001), ML average CoG sway velocity in OL_EO (*F*_(1, 26)_ = 0.615, *p* = 0.44, $\eta_{p}^{2}$ = 0.023), AP average CoG sway velocity in OL_EO (*F*_(1, 26)_ = 4.202, *p* = 0.051, $\eta_{p}^{2}$ = 0.139), ML average CoG sway velocity in OL_EC (*F*_(1, 26)_ = 0.029, *p* = 0.87, $\eta_{p}^{2}$ = 0.001), and AP average CoG sway velocity in OL_EC (*F*_(1, 26)_ = 1.755, *p* = 0.20, $\eta_{p}^{2}$ = 0.063). A significant main effect of time was observed for the AP average CoG sway velocity in OL_EO (*F*_(1, 26)_ = 5.473, *p* = 0.03, $\eta_{p}^{2}$ = 0.174), ML average CoG sway velocity in OL_EC (*F*_(1, 26)_ = 14.103, *p* = 0.001, $\eta_{p}^{2}$ = 0.352), and AP average CoG sway velocity in OL_EC (*F*_(1, 26)_ = 24.281, *p* < 0.001, $\eta_{p}^{2}$ = 0.483), but no main effect of intervention. It was found that the AP average CoG sway velocity in OL_EO, ML average CoG sway velocity in OL_EC, and AP average CoG sway velocity in OL_EC were significantly decreased after the stimulation as compared to pre-stimulation regardless of the two stimulation types (*p* < 0.05). Specifically, the average percent decreases in the AP average CoG sway velocity in OL_EO, ML average CoG sway velocity in OL_EC, and AP average CoG sway velocity in OL_EC following anodal HD-tDCS were 0.8 ± 11.5%, 8.5 ± 11.4%, and 10.7 ± 9.5%, respectively, while the average percent changes following sham stimulation were 9.7 ± 15.2%, 11.0 ± 13.1%, and 17.0 ± 16.5%, respectively (Table 2).

## 4. Discussion

The tDCS procedure has been applied to the treatment and rehabilitation of multiple mental and neurological diseases [33]. However, its effectiveness has not been fully assessed in the field of human movement science, including in the rehabilitation and improvement of foot-related physical performance. In this pilot study, the direction of effects suggested that single-session HD-tDCS may improve the flexor strength of the first toe, although this increase in strength did not significantly differ from sham stimulation. Moreover, participants also showed improvements in the passive ankle kinesthesia threshold and static standing balance performance from pre to post stimulation, while no significant differences were observed between anodal HD-tDCS and sham stimulation. To our knowledge, this is the first study designed to examine the effects of HD-tDCS on foot-related physical performance, demonstrating that tDCS may be a promising method to improve the foot muscle strength and potentially sensation, and could provide novel insights into the potential role of brain cortical regions in the regulation of foot function.

For both athletes and those with diminished foot function, improving foot muscle strength, kinesthesia, and static balance is related to better sports performance and can help the prevention and rehabilitation of injuries and risk events in daily life [34]. Previous studies have provided preliminary evidence that anodal tDCS can improve muscle strength, foot sensory function, and static balance. Tanaka et al. [15] reported that tDCS significantly increased the toe pinch force by stimulating M1, with the observed effect remaining for at least 30 min. Zhou et al. [16] observed that anodal tDCS lowered foot sole vibratory thresholds of the elderly when standing. Studies have also demonstrated that tDCS can improve the postural stability of young adults when standing quietly with TL_EC [35] and enhance the adjustment ability to respond to complex postures [36], indicating that tDCS may be considered as a novel approach to improve foot-related function. However, several other studies showed the opposite results, reporting that tDCS may not significantly improve these functions. Maeda et al. [37], for example, observed that anodal tDCS failed to enhance the lower extremity muscle strength in healthy participants. Similarly, studies also showed that anodal tDCS did not significantly elevate the maximal force production of knee extensors [38] nor enhance static balance ability [39]. In this pilot study, we observed that a significant improvement in passive ankle kinesthesia and static standing balance performance from pre to post intervention was induced by HD-tDCS, which was in line with results from previous studies showing tDCS-induced benefits on physical performance. On the other hand, no statistically significant differences were observed in foot muscle strength, passive kinesthesia threshold, and static balance between the two stimulation types, consistent with the studies showing no significant improvement induced by tDCS.

Several reasons may account for the interesting findings in this study. One is related to potential ceiling effects. In this study, only healthy younger adults were enrolled, and they had excellent physical performance, including high-level muscle strength, great capacity to perceive the trivial changes in ankle motion, and thus great ability to maintain standing balance. Thus, it was possible that the benefit induced by HD-tDCS in physical performance was limited by a “ceiling effect” [16]. Besides, it should also be noted that in addition to sensory-motor regions, other brain regions are also involved in the regulation of the foot strength, sensation, and standing postural sway, such as the prefrontal cognitive regions, insular cortex, and the supplementary motor area. Targeting only one region in this healthy cohort may not be able to induce significant functional improvement.

Meanwhile, though HD-tDCS was used in this study, we know that the brain structure varies across individuals even in healthy younger cohorts, and such inter-subject variance in brain structure may increase the diffusion of the current in the targeted brain regions. Studies have shown that “on-target” current intensity was associated with an increase in functional performance [40]. Therefore, a “personalized” HD-tDCS montage design by using the brain structure MRI data of each individual in combination with advanced neuro-modelling techniques may boost the effects of tDCS interventions on these functional improvements.

Interestingly, although our study had a good blinding effect (35.7%), the INV and DF kinesthesia threshold and the AP and ML average CoG sway velocity in OL_EC were decreased from pre- to post-stimulation both the HD-tDCS and sham groups, and sham stimulation induced similar percent changes in these outcomes compared to HD-tDCS. In this conventional sham control protocol, it was believed only feelings on the scalp similar to those in anodal stimulation would be sensed, but not those of induced cortical activation [41]. However, it was unavoidable that the 30-second stimulation at the beginning of sham would potentially induce certain neurobiological effects on the targeting cortex and lead to improvements in functional performance [42]. A previous study, for example, revealed that event-related electroencephalogram components (P3) related to response time and accuracy were significantly lowered in sham stimulation, and changes in P3 amplitude were moderately correlated with changes in work memory accuracy. This suggested that sham stimulation may have biological effects and alter neuronal function [43]. This may partially explain the effects of sham stimulation we observed here. Novel active sham stimulation has been found to more effectively blind participants and operators to the stimulation condition without affecting functional outcomes [44]. Implementing this new approach in future studies would be worthwhile to help better examine the effects of HD-tDCS on functional performance pertaining to the foot.

To date, the mechanisms by which tDCS might improve physical performance remain largely unclear and the effects of tDCS on physical performance have been found to be inconsistent. The high inter-individual variability, the different electrode montages, and various stimulation protocols (i.e., stimulation types, electrode size and position, intensity, duration) may be contributors to the variable results [13]. Thus, this pilot study may provide some implications for selecting optimal stimulation parameters for future study. Besides, several studies have reported that tDCS applied over the M1 had a positive effect on motor imagery [36], providing some implications in order to explore the beneficial effects of imagery conditions on physical performance during tDCS in future studies [45,46].

There are some limitations in this study. In this pilot study, only a small sample of male participants was enrolled; future studies with a larger sample size of participants with similar numbers of men and women are thus needed. This study focused on only a healthy cohort, and the exploration of the effects of tDCS on the foot function and balance in those with diminished or impaired functionality, such as those with foot injuries, would be worthwhile. It is also necessary to examine the effects of both anodal and cathodal tDCS on cortical activation of the brain and functional performance. This may help to better understand the causal role of brain activity in the regulation of behavior.

## 5. Conclusions

This pilot study was the first to examine the effects of single-session anodal HD-tDCS designed to target the sensory-motor regions of the brain with respect to foot muscle strength, passive ankle kinesthesia, and static balance. The results suggested that single-session HD-tDCS may improve the flexor strength of the first toe, passive ankle kinesthesia, and static standing balance performance, although no significant differences were observed with regard to such effects between anodal HD-tDCS and sham stimulation. This may be potentially due to ceiling effects and the small sample size in this study. Nevertheless, these preliminary findings may inform future studies with larger sample sizes aimed at confirming and expanding the findings of this pilot study by providing knowledge on optimal stimulation parameters, effect size, and power estimation of the tDCS intervention.

## Figures and Tables

**Table 1:** Effects of HD-tDCS on passive ankle kinesthesia and foot muscle strength.

Variables	HD-tDCS		Sham	
	Pre	Post	Pre	Post
PF (°)	1.29 ± 0.46	1.19 ± 0.45	1.38 ± 0.52	1.35 ± 0.39
DF (°)	1.48 ± 0.65	1.36 ± 0.42	1.44 ± 0.53	1.28 ± 0.32
INV (°)	2.73 ± 1.31	2.33 ± 1.15	2.77 ± 1.23	2.44 ± 1.22
EV (°)	2.43 ± 0.61	2.17 ± 0.95	2.37 ± 0.82	2.22 ± 0.79
MPJ flexor strength (N/kg)	1.56 ± 0.53	1.64 ± 0.38	1.43 ± 0.50	1.57 ± 0.49
Flexor strength of the first toe (N/kg)	1.45 ± 0.58	1.61 ± 0.67	1.46 ± 0.58	1.43 ± 0.49
Flexor strength of the other four toes (N/kg)	1.25 ± 0.41	1.30 ± 0.39	1.19 ± 0.41	1.24 ± 0.44
Flexor strength of the all five toes (N/kg)	2.84 ± 0.57	2.80 ± 0.63	2.62 ± 0.54	2.74 ± 0.56

Notes: PF: plantarflexion; DF: dorsiflexion; INV: inversion; EV: eversion; MPJ: metatarsophalangeal joint; HD-tDCS: high-definition transcranial direct current stimulation.

**Table 2:** Effects of HD-tDCS on static balance.

Posture Conditions	Variables	HD-tDCS		Sham	
		Pre	Post	Pre	Post
TL_EO	ML average CoG sway velocity (mm/s)	6.53 ± 1.12	6.60 ± 1.03	6.40 ± 1.06	6.60 ± 1.09
	AP average CoG sway velocity (mm/s)	8.48 ± 1.45	8.41 ± 1.23	8.43 ± 1.40	8.72 ± 1.76
TL_EC	ML average CoG sway velocity (mm/s)	6.64 ± 0.82	7.03 ± 0.97	6.40 ± 1.17	6.46 ± 1.36
	AP average CoG sway velocity (mm/s)	9.40 ± 1.54	9.28 ± 1.43	9.19 ± 2.02	9.01 ± 1.75
OL_EO	ML average CoG sway velocity (mm/s)	31.63 ± 7.28	30.89 ± 7.80	33.42 ± 12.31	31.38 ± 10.52
	AP average CoG sway velocity (mm/s)	29.04 ± 4.65	28.75 ± 5.28	33.63 ± 11.35	29.34 ± 6.55
OL_EC	ML average CoG sway velocity (mm/s)	65.43 ± 15.80	59.56 ± 14.70	65.94 ± 17.23	59.52 ± 18.56
	AP average CoG sway velocity (mm/s)	67.73 ± 14.45	60.20 ± 13.39	71.79 ± 17.05	58.73 ± 13.64

Notes: TL_EO: two-leg standing with eyes open; TL_EC: two-leg standing with eyes closed; OL_EO: one-leg standing with eyes open; OL_EC: one-leg standing with eyes closed; ML: medial–lateral; AP: anteroposterior; HD-tDCS: high-definition transcranial direct current stimulation; CoG: the center of gravity.
